# Xenotransplantation of Human Spermatogonia Into Various Mouse Recipient Models

**DOI:** 10.3389/fcell.2022.883314

**Published:** 2022-05-23

**Authors:** Dongli Liang, Qi Sun, Zijue Zhu, Chuanyun Wang, Shicheng Ye, Zheng Li, Yuan Wang

**Affiliations:** ^1^ Laboratory Animal Center, Instrumental Analysis Center, Shanghai Jiao Tong University, Shanghai, China; ^2^ Shanghai Key Laboratory of Regulatory Biology, Institute of Biomedical Sciences and School of Life Sciences, East China Normal University, Shanghai, China; ^3^ Department of Andrology, The Center for Men’s Health, Urologic Medical Center, Shanghai Key Laboratory of Reproductive Medicine, Shanghai General Hospital, Shanghai Jiao Tong University School of Medicine, Shanghai, China; ^4^ Department of Animal Sciences, College of Agriculture and Natural Resources, Michigan State University, East Lansing, MI, United States

**Keywords:** spermatogonial stem cells, transplantation, germ cells, immunecompetent mouse, human pluripotent stem cells

## Abstract

Spermatogonial stem cells are the foundation of continuous spermatogenesis in adult mammals. Xenograft models have been established to define human SSCs, mostly using infertile and immune-deficient mice as the recipients for human germ cell transplantation. However, it is time-consuming to prepare such recipients using irradiation or chemotherapeutic agents, and this approach may also introduce confounding factors when residual endogenous germ cells recover in transplanted recipients. It remains to be determined whether immune-competent genetically infertile mice can be suitable recipients for xenotransplantation. In this study, we observed similar engraftment efficiencies when using spermatogonia from human biopsied testes across immune-deficient nude mice, immune-competent ICR mice, and genetically infertile *Kit* ^*w/w-v*^ mice, suggesting minimal immunological rejection from immune-competent mouse recipients upon xenotransplantation of human germ cells. More importantly, we derived EpCAM negative and TNAP positive spermatogonia-like cells (SLCs) from human pluripotent stem cells (PSCs), which highly expressed spermatogonial markers including PLZF, INTERGRINα6, TKTL1, CD90, and DRMT3. We found that upon transplantation, these SLCs proliferated and colonized at the basal membrane of seminiferous tubules in testes of both immune-deficient nude mice and *Kit* ^*w/w-v*^ mice, though complete spermatogenesis would likely require supporting human signaling factors and microenvironment. Taken together, our study functionally defined the cell identity of PSC-derived SLCs, and supported xenotransplantation using genetically infertile recipients as a convenient model for functionally evaluating spermatogonia derived from different species.

## Introduction

Spermatogonial stem cells (SSCs) provide a pool of undifferentiated spermatogonia to support continual spermatogenesis in adult mammals and are essential for maintaining male fertility (Oatley and Brinster, 2012). Germ cell transplantation was developed in rodents almost 30 years ago and had since become a gold standard to define functional mouse SSCs (Brinster and Avarbock, 1994; Brinster and Zimmermann, 1994). Two types of recipients are widely used for mouse SSC transplantation. One is irradiation- or chemo-treated wildtype male mice, of which, busulfan is the most commonly used chemotherapeutic agent to ablate endogenous germ cells including SSCs from recipients (Russell and Brinster, 1996; Hermann et al., 2007; Morimoto et al., 2021). However, busulfan rarely eradicates all recipient SSCs, and endogenous spermatogenesis may recover over time and thus complicate precise quantitation of unmarked donor SSCs in recipient testes (Morimoto et al., 2021). The other recipient type is *Kit* ^*w/w-v*^ genetically infertile mice that contain mutations at the white-spotting (W) genomic locus encoding KIT protein (Nocka et al., 1989; Brinster and Zimmermann, 1994; Ohta et al., 2003). Because the tyrosine kinase receptor KIT plays a critical role in spermatogonial development (Yoshinaga et al., 1991; Rossi et al., 2000), *Kit* ^*w/w-v*^ mice with KIT mutations lack endogenous spermatogenesis (Ogawa et al., 2000), which allows accurate quantitation of donor SSCs. Both types of recipients provide a suitable microenvironment for donor mouse SSCs to go through complete spermatogenesis.

Autologous transplantation of human SSCs has been proposed as a strategy to rescue male infertility or restore spermatogenesis in patients after chemotherapy or irradiation treatment in the clinic. In some cases, SSCs collected from fresh testes have to be processed to remove malignant cells, while limited SSCs in cryopreserved testes may need to be expanded before transplantation back to patients (Sadri-Ardekani et al., 2009; Sadri-Ardekani et al., 2011; Koruji et al., 2012; Mirzapour et al., 2012; Jabari et al., 2020). SSC properties and functions could be altered during these procedures, making it necessary to define SSCs *in vivo* before using them for therapeutic applications. Because experimental manipulation involving human subjects is ethically limited, animal xenotransplantation provides a powerful approach to understand the properties of human SSCs. So far, xenotransplantation has been reported using donor germ cells from primates, humans, and many other species (Jiang and Short, 1995; Honaramooz et al., 2002; Nagano et al., 2002; Honaramooz et al., 2003; Hermann et al., 2007; Sadri-Ardekani et al., 2009; Sadri-Ardekani et al., 2011; Kubota and Brinster, 2018; Morimoto et al., 2021). Although human SSCs cannot differentiate and complete spermatogenesis in mouse testes, they do transiently colonize and proliferate at the basement membrane of mouse seminiferous tubules (Nagano et al., 2002; Sadri-Ardekani et al., 2009; Sadri-Ardekani et al., 2011). Notably, published studies mainly used busulfan-treated immunocompromised nude mice as xenotransplantation recipients (Nagano et al., 2002; Sadri-Ardekani et al., 2009; Sadri-Ardekani et al., 2011), though it is unclear whether immunodeficiency may enhance the survival of donor human SSCs in mouse testes. It remains to be determined whether immune-competent or genetically infertile mice (e.g., *Kit* ^*w/w-v*^) are suitable recipients for xenotransplantation.

In addition to using SSCs from testes to rescue male infertility, recent technical advances make it possible to derive germ cells from pluripotent stem cells (PSCs) for future therapy of male infertility. PSCs include embryonic stem cells (ESCs) that are derived from embryonic blastocysts (Evans and Kaufman, 1981; Martin, 1981; Thomson et al., 1998), and induced PSCs (iPSCs) from somatic cells (Takahashi and Yamanaka, 2006; Takahashi et al., 2007; Yu et al., 2007; Park et al., 2008). Differentiation protocols have been developed to direct human PSCs into primordial germ cells, spermatogonia-like cells (SLCs) and/or haploid spermatogenic cells (Kee et al., 2009; Easley et al., 2012; Irie et al., 2015; Sasaki et al., 2015; Zhao et al., 2018; Gell et al., 2020). These, coupled with breakthroughs of iPSC research in the last 2 decades, may eventually make it possible to derive autologous germ cells from patient iPSCs for replacement therapy. Yet it is still unknown whether these PSC-derived SLCs behave similarly in testes as *in vivo* developed spermatogonia. Additionally, in treating male infertility using replacement therapy, potential immune responses elicited by PSC-derived spermatogonia need to be considered. Although reports showed minimal immune rejection upon transplantation of syngeneic PSC-derived cells or tissues (Araki et al., 2013; Guha et al., 2013), the immunogenicity of these cells remains to be a highly debatable topic, and may vary by cell types (de Almeida et al., 2013; Liu et al., 2017). Therefore, it is necessary to examine the survival and tolerance of PSC-derived spermatogonia by the host *in vivo*.

In this study, we assessed the engraftment of human spermatogonia derived from different sources in immune-competent and immunocompromised mouse recipients. In addition, by using the xenotransplantation models, we examined the cell identity of EpCAM-/TNAP+ SLCs differentiated from human PSCs. We further demonstrated that xenotransplantation with *Kit* ^*w/w-v*^ genetically infertile mice as recipients provided a convenient model to functionally evaluate human spermatogonial property *in vivo*.

## Materials and Methods

Overall experimental design was shown below. Briefly, we used two types of human spermatogonia for xenotransplantation, one from biopsied human testes, the other from SLCs collected from differentiated PSCs. Three types of recipients were used, including immune-deficient nude mice, immune-competent ICR mice, and genetically infertile *Kit* ^*w/w-v*^ mice, as listed on the right of the graph.
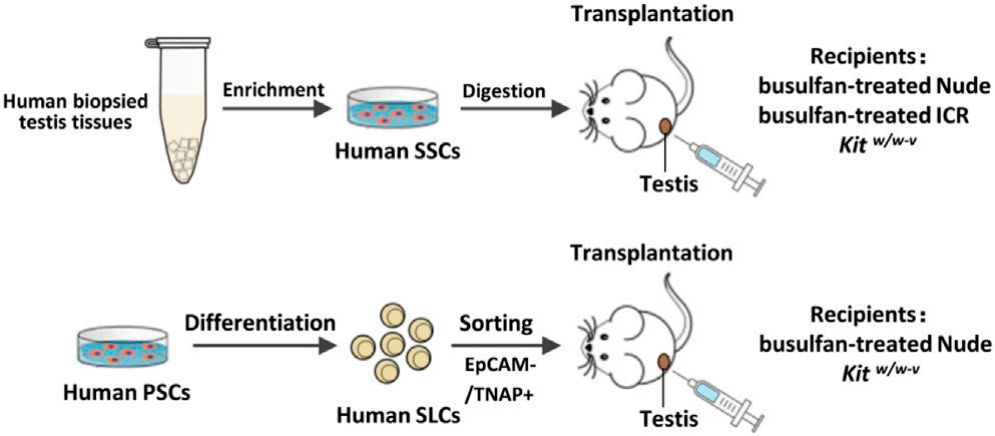


### Collection of Male Germ Cells From Human Testicular Tissues

Testicular specimens were biopsied from obstructive azoospermia (OA) patients and immediately placed aseptically into Dulbecco’s modified Eagle’s medium (DMEM, Gibco) containing 1,000 U/ml penicillin and streptomycin (PS, Gibco) for further processing. The diagnosis of OA patients was confirmed by pathological examination. This study was approved by the Institutional Ethical Review Committee of Shanghai First People’s Hospital (license number 2016KY196), Shanghai Jiao Tong University School of Medicine. All participants had provided written consents. In total, eight testis samples were collected from OA patients from age 25 to 40. Collected testis tissues were cut into small pieces and placed in the cryopreservation medium consisting of DMEM, 20% fetal bovine serum (FBS, Hyclone), 10% dimethyl sulfoxide (DMSO, Sigma-Aldrich), and stored in liquid nitrogen. For experiments, the testis tissues were rapidly thawed at 37°C and washed in DMEM. Recovered testicular samples were processed with a two-step enzymatic digestion, as described previously (Hermann et al., 2007; Hermann et al., 2009). Briefly, testicular tissues were first digested with collagenase type IV, followed by digestion with 0.25% trypsin, 0.38 g/L of EDTA, and 1.4 mg/ml DNase I for 5 min. The digestion was stopped by adding DMEM with 10% FBS, and dissociated single cells were collected by centrifugation. Cell suspensions were seeded into gelatin-coated culture plates in DMEM/F-12 (Gibco) supplemented with 10% FBS for 24–36 h, according to a procedure previously described (Wu et al., 2009). More than 95% of somatic cells (including Sertoli cells, Leydig cells, and peritubular cells) were attached to culture plates, while non-adherent germ cells were collected by centrifuge. The germ cells were then seeded into laminin-coated culture plates in DMEM/F12 containing 1x PS, 6 mM l-Glutamine (Gibco), 100 μM β-mercaptoethanol (Sigma-Aldrich), 1xB27 (Gibco), 20 ng/ml human GDNF (Sino Biological), and 20 ng/ml human basic fibroblast growth factor (b-FGF, Sino Biological) for 2 days at 34°C in a humidified 5% CO_2_ incubator. These freshly isolated laminin-binding cells were enriched with SSCs and spermatogonia. In total, ∼5×10^5^ human SSCs and spermatogonia were obtained from 8 cryopreserved biopsied samples. After 2-days culture, about 30,000 human germ cells per testis were injected for xenotransplantation.

### Animal Experiments and Xenotransplantation

Male nude mice (BALB/c-*nu/nu*) and ICR mice at 6 weeks were injected intraperitoneally with 40 mg/kg busulfan (Sigma-Aldrich) and were subsequently used as recipients 6 weeks later. Male *Kit* ^*w/w-v*^ mice at 12-week old were used as recipients. All animals used for this study were housed at an SPF-graded facility with healthy conditions. Germ cells to be transplanted were suspended in ∼10 μl PBS with 10% trypan blue (v:v; Invitrogen) and injected into the seminiferous tubules of recipient testes *via* the efferent ducts. The contralateral testis in the same mouse with mock injection with PBS and trypan blue was used as a control. PBS was used to minimize any potential effects of proteins, nutrients, or small molecules in the culture media on germ cell proliferation and development. Six weeks after transplantation, animals were euthanized, and their testes were removed for further analyses. All animal experimental procedures were conducted in accordance with the local Animal Welfare Act and Public Health Service Policy with approval from the Committee of Animal Experimental Ethics at East China Normal University (Ref #:M20170325).

### Histology, Immunohistofluorescence and Immunofluorescence Assays

Histology and IHF were performed as previously described (Zhao et al., 2018). Briefly, mouse testis samples were fixed with 4% PFA solution, paraffin-embedded, and sectioned with 4 μm thickness. Following the antigen retrieval by citrate (pH6.0, boiling for 15–20 min and cooling down for 30 min), testis sections were blocked with 1% goat serum (Abcam, ab7481) in PBS at 4°C for 12–16 h, stained with primary antibodies at 4°C for 12–16 h, washed three times (15–30 min each time) with PBS at room temperature, and then stained with goat anti-rabbit IgG AlexaFluor 568 (Invitrogen) and goat anti-mouse IgG AlexaFluor 488 (Invitrogen) at 4°C for 12–16 h, and washed three times (15–30 min each time) in dark with PBS at room temperature. Primary antibodies used in this study: mouse anti-DDX4 (Abcam, ab27591), rabbit anti-DDX4 (Abcam, ab13840), rabbit anti-NuMA (Novus Biologicals, NB100-74636), rabbit anti-GFRα1 (Abcam, ab8026), mouse anti-PCNA (Abcam, ab29), and mouse anti-PLZF (Santa Cruz, sc-28319). The fluorescein-conjugated secondary antibodies were used at 1:300 dilution. Images were obtained with a Leica confocal microscope.

For IF, cells cultured on gelatin-coated coverslips were washed twice with 1× PBS and fixed in 4% PFA for 20 min at room temperature. Alternatively, cells were dissociated by Trypsin (Gibco), placed onto slides by cytospin preparation and fixed in 4% PFA for 20 min at room temperature. IF was performed as previously described (Zhao et al., 2018). Briefly, after treatment with 0.2% Triton-100 for 15 min, fixed cells were stained with primary antibodies at 4°C for 12–16 h, washed three times with PBS (10 min every time), and then stained with goat anti-rabbit IgG AlexaFluor 568 (Invitrogen) at 1:300 dilution at 4°C for 12–16 h, and washed three times (10 min each time) in the dark with PBS at room temperature. Primary antibodies used in this study: DDX4 (Abcam, ab13840), GPR125 (GeneTex, GTX51219), and PLZF (R&D, MAB2944). Images were obtained with a Leica fluorescent microscope.

### Human PSC Culture and Differentiation

All PSC culture and differentiation were performed as previously described (Zhao et al., 2018). Briefly, human embryonic stem cell line H1 (WiCell) was maintained in chemically defined Essential 8 medium (Stem Cell Technologies). Cells were passaged every 5–7 days and dissociated by 1 mg/ml Collagenase IV (Millipore). ESCs were induced when reaching 80–90% confluence in differentiation medium [α-MEM (Gibco) containing 2 mM l-glutamine (Gibco), 1× Insulin-Transferrin-Selenium-X (Gibco), 0.2% KnockOut SR XenoFree CTS (Gibco), 1 ng/ml human b-FGF, 20 ng/ml human GDNF (Sino Biological), 0.2% chemically defined lipid concentrate, and 200 μg/ml vitamin C (Sigma)] (Zhao et al., 2018). Medium was changed every day. No passage of cells was performed during differentiation. By day 12, most cells were PLZF+ SLCs. SLCs were collected for analyses or sorted for transplantation on day 12 of ESC differentiation.

### Flow Cytometry Analysis

Mouse peripheral blood samples were obtained *via* tail tip into anticoagulant EDTA-containing tubes. After being treated with red blood cell lysis buffer (Beyotime Biotechnology), blood cells were pelleted at 1,200 rpm for 15 min and re-suspended in DMEM with 10% FBS, followed by incubation with PE-CD3 (Cat. #: 561824), PE-Cy7-CD8 (Cat. #: 561097), FITC-CD4 (Cat. #: 561828) or FITC-CD19 (Cat. #: 561740) antibodies from BD Pharmingen. Subsequently, cells were washed with DMEM and analyzed with a BD Fortessa analyzer (BD Biosciences).

Cells at day 12 of PSC differentiation were prepared for flow cytometry analyses as described previously (Zhao et al., 2018). Briefly, cells were dissociated by TrypLE (Gibco), fixed by 4% PFA for 20 min at room temperature, suspended in FACS buffer (PBS with 5% fetal calf serum, 0.2% Triton-100, and 0.5% Tween 20), and centrifuged at 500×g for 5 min before incubation with antibodies. Antibodies used for examining PSC-derived SLCs: EpCAM (Biolegend, 324203), TNAP (Biolegend, 327305), TRA-1-81 (Santa Cruz, Sc-21706), INTEGRINα6 (Biolegend, 313615) and PLZF (R&D, MAB2944). Subsequently, cells were washed with DMEM and analyzed with a BD Fortessa analyzer (BD Biosciences).

To collect SLCs from *in vitro* differentiated PSCs by flow cytometry, dissociated cells were stained at 4°C for 45–60 min in DMEM containing antibodies and then sorted by a FACS Aria II (BD Biosciences). Antibodies used for SLC sorting: EpCAM (Biolegend, 324203), TNAP (Biolegend, 327305) and CD90 (Abcam, ab133350).

### RT-PCR and Quantitative Real-Time PCR

Total RNAs were extracted with Trizol (Thermo Fisher Scientific) and cDNAs were synthesized using a PrimeScript^®^ RT reagent Kit (TaKaRa) following manufacturer’s protocols, as previously described (Zhang et al., 2016). *Gapdh* was used as the house-keeping gene to normalize various sorted cell populations. The primer sequences used in this assay are provided in Supplementary Table S1.

### Statistical Analysis

Data were presented as mean ± standard error (SEM). Unpaired Student’s t-test was conducted to examine between-group differences using GraphPad Prism. All experiments were performed independently for more than three times unless otherwise stated. *: *p* < 0.05; **: *p* < 0.01.

## Results

### Characterize Human Spermatogonia From Obstructive Azoospermia Patients

**FIGURE 1:**
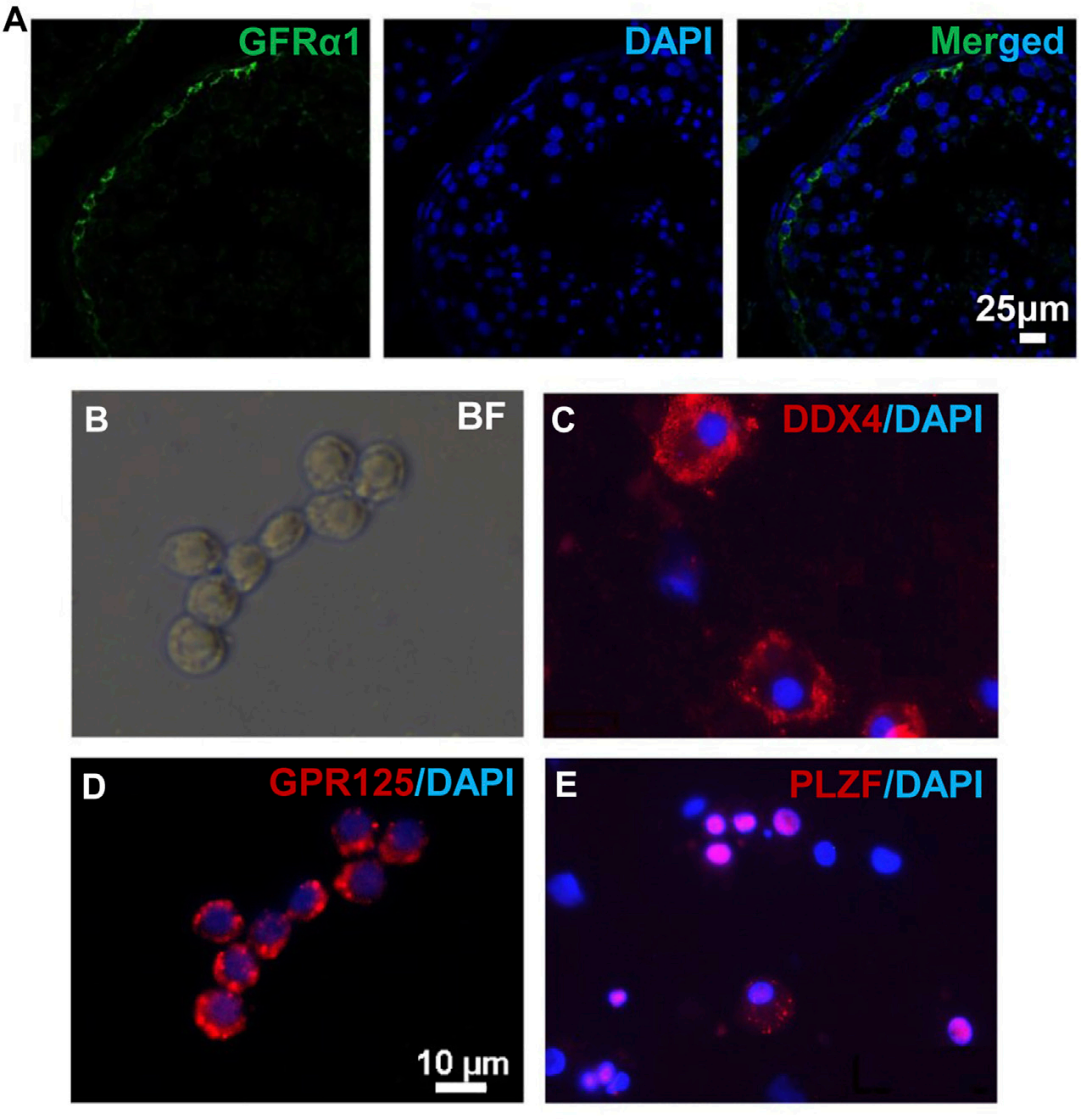
Human germ cells were collected from biopsied testes. **(A)** IHF was performed on biopsied testes from OA patients with a GFRα1 antibody. **(B–E)** Isolated human spermatogonia were observed in bright field **(B)**, and detected by IF with antibodies against DDX4 **(C)**, GPR125 **(D)**, and PLZF **(E)**, counterstained with DAPI. Scale bar: 10 μm **(B,D)** cells were cultured on a cover glass. **(C,E)** cells were spun onto slides by cytospin preparation.

To obtain *in vivo* developed human spermatogonia, human testicular samples were biopsied from obstructive azoospermia (OA) patients, and the existence of undifferentiated spermatogonia in these samples was confirmed by immunohistofluorescence (IHF) with an antibody against a SSC marker GFRα1 (Figure 1A). Biopsied testis tissues were processed with two-step enzymatic digestion as described previously (Hermann et al., 2007; Hermann et al., 2009). Non-adherent germ cells were separated from somatic supporting cells (e.g., Sertoli cells) that were attached on gelatin-coated plates. These germ cells were further seeded onto laminin-coated dishes (Figure 1B) and cultured for 2 days to enrich for spermatogonia before immunofluorescence (IF) assay and transplantation. More than 95% (based on 10 fields of view) of the enriched cell population was stained with an antibody against a germ cell-specific protein, DDX4, supporting their germ cell identity (Figure 1C). In addition, these cells contain ∼80% undifferentiated spermatogonia (based on 10 fields of view), as labeled by the high expression of SSC markers, GPR125 (Figure 1D) and PLZF (Figure 1E) (Costoya et al., 2004; Seandel et al., 2007; He et al., 2012). In total, human germ cells were collected from eight OA patients.

### Characterize Infertile Mouse Models for Xenotransplantation of Human Germ Cells

**FIGURE 2:**
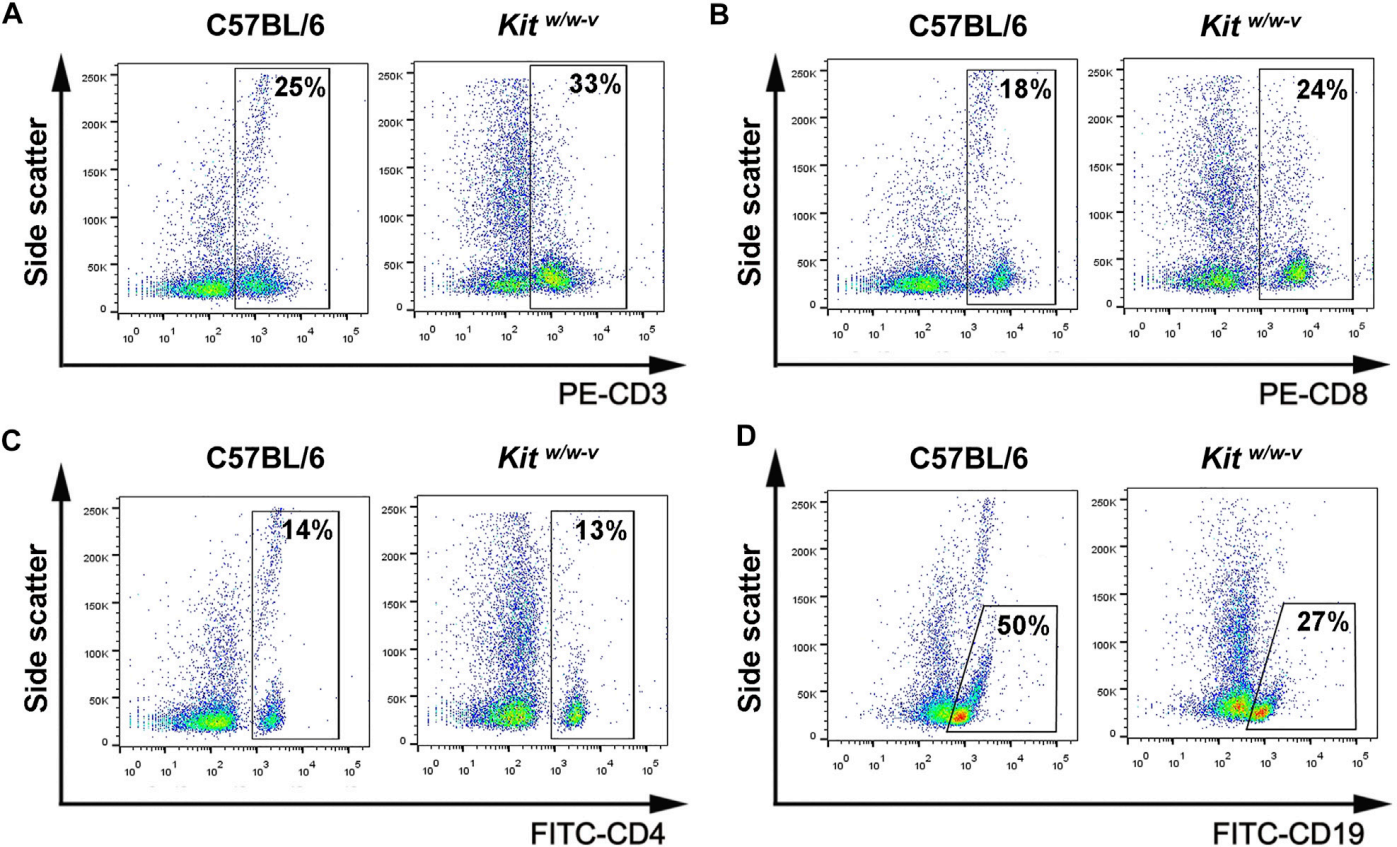
*Kit* ^*w/w-v*^ mice displayed similar peripheral lymphocyte composition, compared to C57BL/6J immune-competent wildtype mice. **(A**–**D)** The percentages of CD3^+^, CD8^+^, and CD4^+^ T lymphocytes, as well as CD19 ^+^ B lymphocytes from peripheral blood from *Kit* ^*w/w-v*^ and C57BL/6 mice were determined with flow cytometry.

To explore whether the immune deficiency enhances the engraftment of human spermatogonia, three mouse strains, including nude mice, ICR, and *Kit* ^*w/w-v*^ male mice, were used as xenotransplantation recipients. Busulfan-treated nude mice (Supplementary Figure S1A) and ICR mice (Supplementary Figure S1B) represent immunocompromised and immune-competent recipients, respectively. The ablation effects of endogenous germ cells in these mice by busulfan were confirmed by histology at week 6 post-treatment (Supplementary Figure S1A, B). Preparation of infertile mouse recipients using busulfan is time-consuming and may potentially introduce confounding factors when residual endogenous germ cells are left or recovered in recipients. Therefore, *Kit* ^*w/w-v*^ genetically infertile mice, which lack endogenous spermatogenesis (Nocka et al., 1990; Reis et al., 2000), were also tested as potential xenotransplantation recipients. Although KIT plays a crucial role in lymphocyte development (Rodewald et al., 1997; Waskow et al., 2002), we observed comparable percentages of CD3^+^ T cells (Figure 2A), CD8^+^ T cells (Figure 2B), and CD4^+^ T cells (Figure 2C), with moderately decreased CD19 ^+^ B cells (Figure 2D) in *Kit* ^*w/w-v*^ mice with those in C57BL/6J wildtype mice (Supplementary Figure S1C), suggesting that *Kit* ^*w/w-v*^ mice contain intact adaptive immunity.

### Human Germ Cells From OA Patients Readily Colonize Both Immune-Competent and Immune-Deficient Infertile Mouse Recipients

About 30,000 human germ cells from OA patients were injected per testis for xenotransplantation. The contralateral testis in the same mouse with mock injection was used as a control. In total, we injected 5 nude mice and 5 ICR mice, both busulfan-treated, as well as 6 *Kit* ^*w/w-v*^ mice. Six weeks after transplantation, recipient testes were dissected for histology and IHF. We found germ cells in about 3% seminiferous tubules in ∼60% of recipient mice (3 for nude and ICR mice, and 4 for *Kit* ^*w/w-v*^ mice) with human spermatogonial injection, and some of them were colonized at the basal membranes (Supplementary Figure S2). By contrast, no germ cells were detected in the contralateral testes with mock injection (Supplementary Figure S2).

**FIGURE 3:**
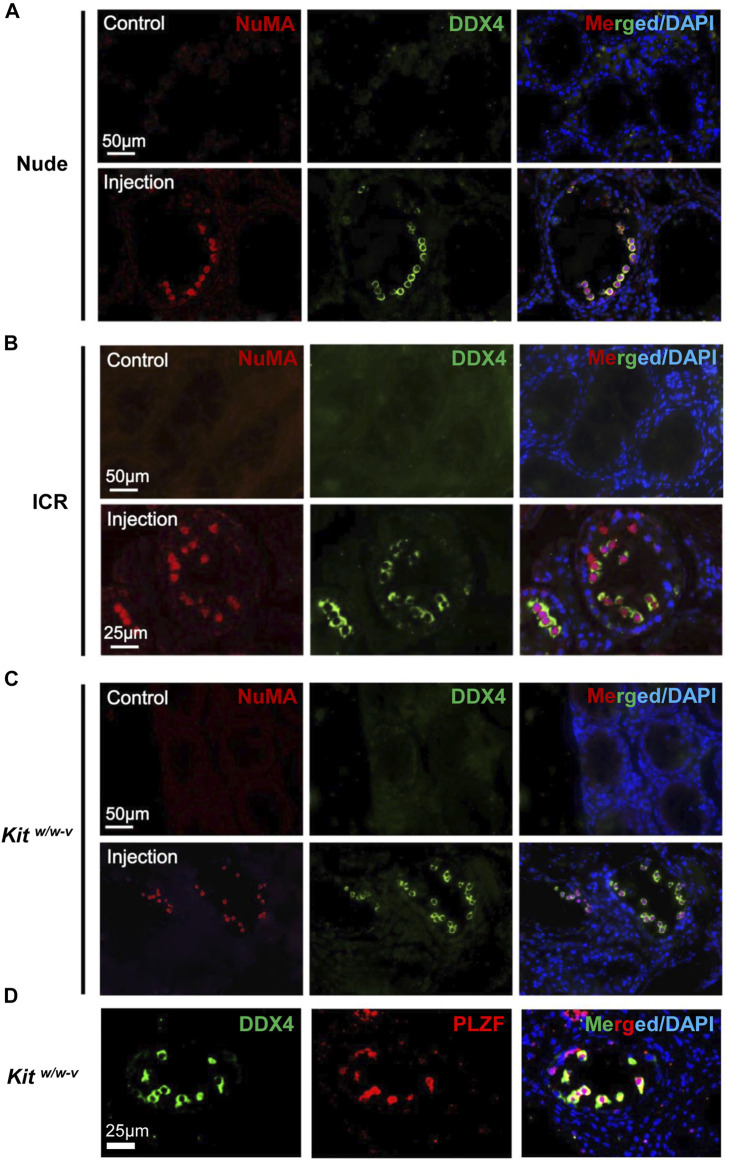
*In vivo* developed human spermatogonia engrafted into testes from both immune-deficient and immune-competent mice. **(A**–**C)** IHF assays were performed on nude, ICR, and *Kit* ^*w/w-v*^ testes at 6 weeks post transplantation, with NuMA and DDX4 antibodies, co-stained with DAPI. **(D)** IHF assays were conducted on injected *Kit* ^*w/w-v*^ testes with antibodies against PLZF and DDX4, counterstained with DAPI. Nude and ICR mice at age 6 weeks were injected intraperitoneally with 40 mg/kg busulfan and were subsequently used as recipients after 6 weeks. *Kit* ^*w/w-v*^ mice at 12-week old were used as recipients. About 30,000 human germ cells from OA patients were injected per testis for xenotransplantation. The contralateral testis in the same mouse with mock PBS injection was used as a control. In total, 5 nude mice, 5 ICR mice, and 6 *Kit* ^*w/w-v*^ mice were injected.

It has reported that endogenous spermatogenesis may recover from busulfan treatment (Morimoto et al., 2021), and occasionally, residue spermatogonia may also exist in *Kit* ^*w/w-v*^ testes (Kubota et al., 2009). To assess the origin of germ cells in these transplanted mice, NuMA (Merdes and Cleveland, 1998; Taimen et al., 2004), an antibody against human cells, was used. We confirmed that NuMA could specifically detect human but not mouse cells by Western blotting, IHF, and IF (Supplementary Figure S3). We performed IHF with this NuMA antibody and an antibody against DDX4, a germ cell-specific protein (Tanaka et al., 2000; Castrillon et al., 2000). No DDX4+/NuMA+ cells were detected in control testes from busulfan-treated nude mice. By contrast, multiple DDX4+/NuMA+ cells were clearly observed at the basal membrane of the contralateral testes from the same mice with transplantation of human germ cells (Figure 3A, Supplementary Figure S4A). Similar results were observed in both busulfan-treated ICR mice (Figure 3B, Supplementary Figure S4B) and *Kit* ^*w/w-v*^ mice (Figure 3C, Supplementary Figure S4C). Because only undifferentiated spermatogonia would survive long-term in mouse testes, these DDX4+/NuMA+ germ cells were likely human spermatogonia. Indeed, all DDX4+ cells also highly expressed the SSC marker, PLZF (Costoya et al., 2004) (Figure 3D). We did not observe significant difference in the number of colonized spermatogonia across these three types of recipients, suggesting that successful engraftments can be achieved with germ cells developed *in vivo* from human testes using both immune-competent and immune-compromised recipients.

### Derivation of SLCs From Human PSCs

In a previous study, we robustly derived SLCs from human PSCs (Zhao et al., 2018). These SLCs highly express human spermatogonia-specific genes, including PLZF, CD90, GPR125, DRMT1, and DMRT3 (Zhao et al., 2018). We thus further investigated whether these *in vitro* PSC-derived SLCs behave similarly to *in vivo* developed spermatogonia from human testes. However, upon injection into testes of busulfan-treated nude mice with whole PSC differentiated population, residual undifferentiated PSCs often formed teratoma (data not shown). To exclude contaminating PSCs in transplanted populations, we need to identify appropriate surface antigens to collect SLCs with flow cytometry.

**FIGURE 4:**
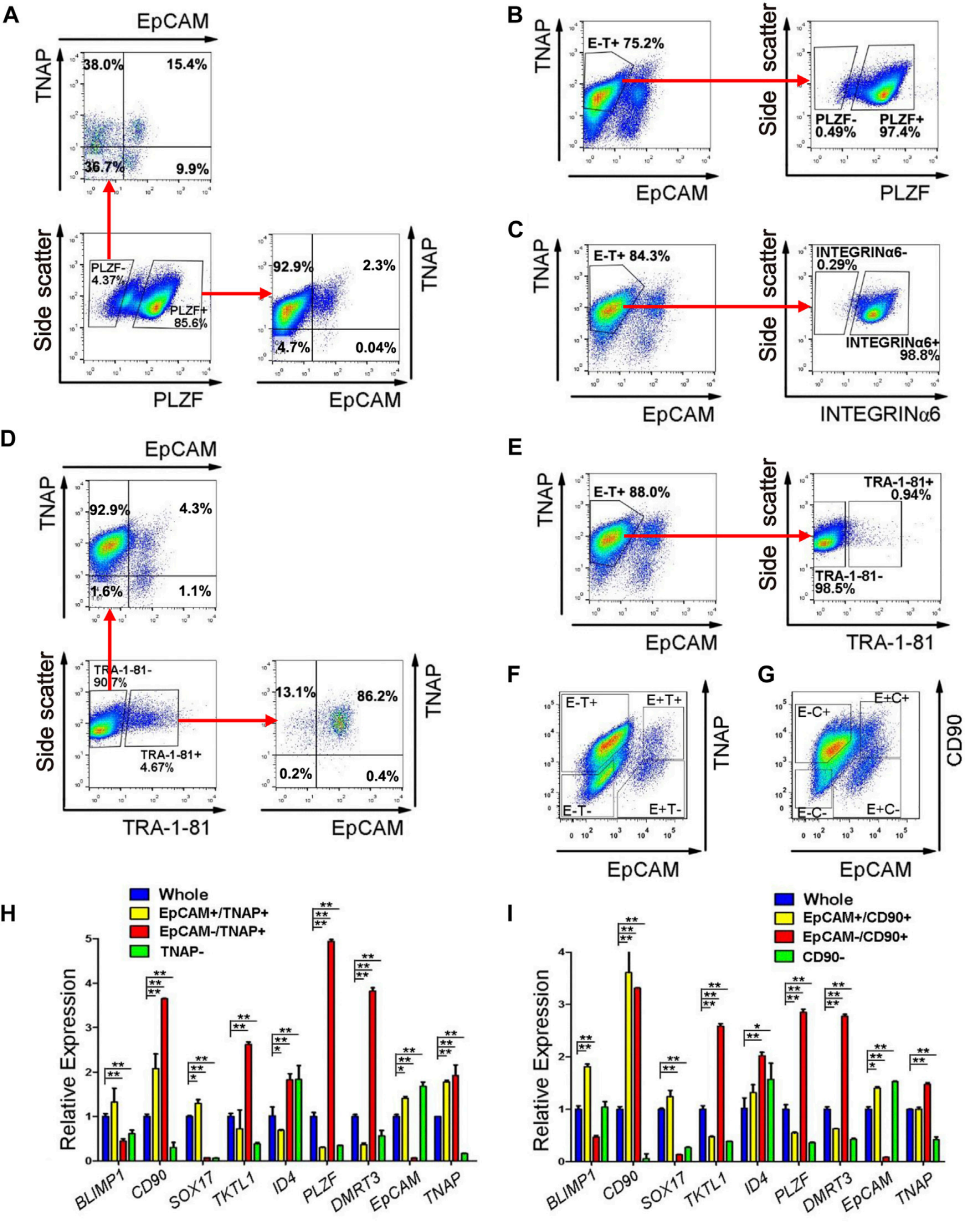
EpCAM-/TNAP+ population is enriched with SLCs differentiated from human PSCs. **(A)** Flow cytometry analyses of EpCAM and TNAP expression in PLZF positive and negative populations from PSCs. **(B,C,E)** Percentage of PLZF **(B)**, INTEGRINα6 **(C)**, and TRA-1-81 **(E)** in EpCAM-/TNAP+ population differentiated from human PSCs, analyzed by flow cytometry. **(D)** Flow cytometry analyses of EpCAM and TNAP expression in TRA-1-81 positive and negative populations from PSCs. **(F–G)** Fluorescence-activated cell sorting was conducted, using EpCAM and TNAP combination **(F)**, or with EpCAM and CD90 antibodies **(G)**. E, EpCAM; T: TNAP; C: CD90 **(H–I)** Real-time RT-PCR analyses of germ cell-specific genes on populations sorted with EpCAM and TNAP **(H)** or with EpCAM and CD90 staining **(I)**. Data represent as the mean ± SEM (n ≥ 3). *: *p* < 0.05; **: *p* < 0.01. Negative gating controls were shown in Supplementary Figure S5.

In our SLCs derived from PSCs, PLZF is the most prominently expressed spermatogonial protein (Zhao et al., 2018). Because PLZF is a transcription factor and located in the nucleus, it cannot be used for live-cell sorting. However, we could use PLZF as a marker for spermatogonia to identify surface antigens that are enriched in SLCs but not in PSCs with flow cytometry on fixed cells. Because TNAP (tissue non-specific alkaline phosphatase) and EpCAM were reported to be highly expressed in primordial germ cells and pro-spermatogonia (Sasaki et al., 2015), we analyzed their co-existence with PLZF in SLCs differentiated from a human ESC line, H1 (Supplementary Figure S5). We found that the majority (92.9%) of PLZF+ SLCs were stained as EpCAM negative (-) and TNAP positive (+) (Figure 4A). In addition, 97.4% of EpCAM-/TNAP+ cell population from differentiated PSCs were positive for PLZF (Figure 4B) and INTEGRINα6 (Figure 4C), a common spermatogonial marker in mouse, monkey and human (Shinohara et al., 1999; Maki et al., 2009; He et al., 2010). Further, TRA-1-81 is a marker for undifferentiated PSCs. We found that 86.2% of TRA-1-81 positive cells were co-stained with EpCAM+/TNAP+ population, while 92.9% of TRA-1-81 negative cells, which lost pluripotency, were EpCAM-/TNAP+ (Figure 4D). These data suggest that we may use EpCAM-/TNAP+ to enrich SLCs and to exclude TRA-1-81 + undifferentiated PSCs (Figure 4E).

We further evaluated the gene expression of cell populations separated by differential EpCAM and TNAP expressions (Figure 4F) with real-time RT-PCR assays. Because CD90 was reported to be spermatogonial marker in mice, primates and humans (Kubota et al., 2003; Maki et al., 2009; He et al., 2010), we also analyzed the cells sorted by EpCAM and CD90 staining (Figure 4G). We confirmed that the transcript level of *EpCAM* was dramatically lower while *TNAP* or *CD90* was significantly upregulated in both EpCAM-/TNAP+ and EpCAM-/CD90+ populations, compared to those from the whole differentiated PSC populations (Figures 4H,I), supporting reliable sorting results. We also observed that the expression of *TKTL1*, *PLZF*, and *DMRT3*, markers specific for spermatogonia, were significantly elevated in both EpCAM-/TNAP+ and EpCAM-/CD90+ SLCs, with a relatively higher PLZF transcript level in EpCAM-/TNAP+ population (Figures 4H,I). These data support the usage of EpCAM-/TNAP+ population to represent PLZF+ SLCs derived from PSCs.

### Human PSC-Derived SLCs Colonize Both Immune-Deficient Nude Mice and Genetically Infertile Mouse Recipients

**FIGURE 5:**
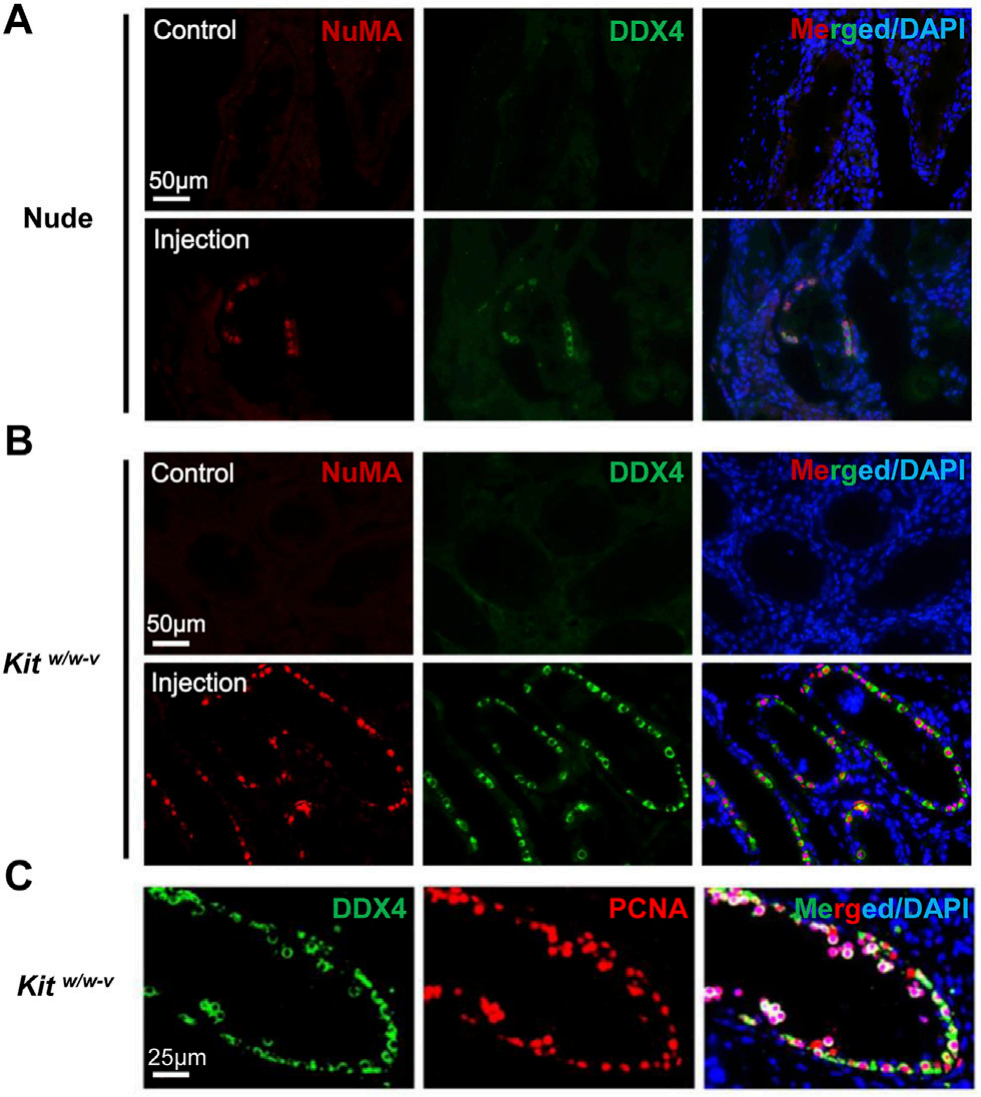
Human SLCs from PSCs readily colonize both nude and *Kit* ^*w/w-v*^ mouse testes. **(A,B)** IHF assays were performed 6 weeks post-transplantation on testes from nude mice and *Kit* ^*w/w-v*^ recipients with antibodies against NuMA and DDX4, counterstained with DAPI. **(C)** IHF assays on testes from *Kit* ^*w/w-v*^ mice with antibodies against PCNA and DDX4, counterstained with DAPI. Nude mice at age 6 weeks were injected intraperitoneally with 40 mg/kg busulfan and were subsequently used as recipients after 6 weeks. Male *Kit* ^*w/w-v*^ mice at 12-week old were used as recipients. About ∼100,000 EpCAM-/TNAP+ SLCs were injected per testis for xenotransplantation. The contralateral testis in the same mouse with mock PBS injection was used as a control. In total, 4 nude mice and 6 *Kit* ^*w/w-v*^ mice were injected.

We next injected ∼100,000 EpCAM-/TNAP+ SLCs per testis into seminiferous tubules of *Kit* ^*w/w-v*^ mice and busulfan-treated nude mice. The contralateral testis from the same mouse was injected with PBS as a control. No teratoma were found in any of 6 *Kit* ^*w/w-v*^ mice and 4 busulfan-treated nude mice we transplanted, indicating that these SLCs lost pluripotency during PSC differentiation. We found no DDX4+/NuMA+ human germ cells in the control testes of transplanted recipients by IHF. By contrast, in 2 nude mice and all 6 *Kit* ^*w/w-v*^ recipients, DDX4+/NuMA+ germ cells were clearly observed at the basement membrane of the other testis from the same mouse 6 weeks post-injection of EpCAM-/TNAP+ SLCs (Figure 5A). Interestingly, compared to engrafted nude mice (with ∼4% of seminiferous tubules containing human germ cells), much more DDX4+/NuMA+ cells were detected in *Kit* ^*w/w-v*^ testes with ∼20% human germ cell-containing seminiferous tubules (Figure 5B, Supplementary Figure S6, 7), suggesting that *Kit* ^*w/w-v*^ mice provided a suitable microenvironment for the survival of human PSC-derived spermatogonia. In addition, these DDX4+ germ cells in *Kit* ^*w/w-v*^ testes displayed high expression levels of the proliferation marker, proliferating cell nuclear antigen (PCNA) (Figure 5C), suggesting that these human SLCs could go through mitotic division in mouse testes. Taken together, our data provide evidence that SLCs derived from human PSCs are indeed undifferentiated spermatogonia, and they maintain their potential to proliferate and repopulate mouse testes upon xenotransplantation.

## Discussion

Previous studies mainly used immune-deficient mouse recipients for human spermatogonial xenotransplantation (Nagano et al., 2002; Sadri-Ardekani et al., 2009; Sadri-Ardekani et al., 2011). However, endogenous spermatogenesis that is ablated by irradiation or chemotherapeutic agents may recover over time in these mice, making it difficult in quantifying transplanted germ cells. It remains to be determined whether immune-competent mice or *Kit* ^*w/w-v*^ genetically infertile mice are suitable recipients for xenotransplantation studies. Here, we systematically evaluated engraftments of human spermatogonia in immunocompromised nude mice, immune-competent ICR mice, and *Kit* ^*w/w-v*^ mice. We found that human spermatogonia settled at the basement membrane in seminiferous tubules of all three types of recipients. In addition, the survival and engraftment efficiency of PSC-derived SLCs were much better in *Kit* ^*w/w-v*^ recipients than those in busulfan-treated nude mice. Our data thus support *Kit* ^*w/w-v*^ mice as ideal recipients to assess spermatogonial properties in xenotransplantation.

Notably, nude mice are immunocompromised, lack T cells, and have reduced NK cell activities (Groscurth and Kistler, 1975; Schedi et al., 1975). By contrast, we did not find significant defects in peripheral T and B lymphocyte composition in *Kit* ^*w/w-v*^ mice, compared to wildtype C57B6/J mice, suggesting that adapted immunity in *Kit* ^*w/w-v*^ mice is largely intact. So why PSC-derived SLCs would survive better in *Kit* ^*w/w-v*^ mice? One possibility is that *Kit* ^*w/w-v*^ mice had markedly reduced mast cells (Gregory et al., 2005; Grimbaldeston et al., 2005), while nude mice have normal counts of mast cells (Mayrhofer and Bazin, 1981; Wlodarski et al., 1983). Increased mast cells in nude mice under certain pathological conditions were also reported (Wlodarski et al., 1983). Mast cells are known to participate in both allergic and inflammatory responses to pathogens (Gilfillan and Tkaczyk, 2006; Kolkhir et al., 2021). When abnormally elevated, mast cells may cause tissue fibrosis and sclerosis in the testis, abnormal spermatogenesis, and male infertility (Jezek et al., 1999; Meineke et al., 2000; Fijak and Meinhardt, 2006). It will be interesting to investigate whether low mast cell counts in *Kit* ^*w/w-v*^ protect PSC-derived SLCs from immunological rejection during xenotransplantation.

In a previous study, Reis et al. did not find human germ cells in any recipient (either *Kit* ^*w/w-v*^ mice and SCID mice) post xenotransplantation (Reis et al., 2000). Later, Nagano et al. demonstrated successful colonization of human spermatogonia in busulfan-treated nude mice (Nagano et al., 2002). Although it was unclear why the mouse recipients used in the Reis et al. study failed to support the xenotransplantation of human germ cells, it has been once attributed to potential interspecies non-compatible cell adhesion molecules and/or immunological rejection (Reis et al., 2000). However, these speculations have never been experimentally examined, and it remains elusive whether immune deficiency of recipients improves the survival and engraftment efficiency of transplanted germ cells. Our data demonstrated that human spermatogonia engrafted into both immune-deficient nude mice and immune-competent ICR mice with similar efficiencies. SSCs and spermatogonia reside at the basal compartment of the seminiferous tubules, lying outside of the blood-testis-barrier (BTB). Thus, SSCs and spermatogonia are physically protected from immunological cells/factors only by basement lamina, peritubular myoid cells (PMCs), and endothelial cells of testicular blood vessels (Qu et al., 2020). However, the immune privilege of testis is established not only by BTB, but also by immune-suppressive factors secreted from testicular supporting cells (e.g., Sertoli cells, Leydig cells, and PMCs) (Kaur et al., 2014; Qu et al., 2020). Tolerance of xenogeneic SSCs and spermatogonia may be induced by local immune-suppressive microenvironment in recipient testis. Consistent with our findings, published reports showed that rat spermatogonia went through complete spermatogenesis in busulfan-treated immune-competent mouse testes (Qu et al., 2012). Although busulfan itself has immune-suppressing effects, the immune system of recipients appeared to recover at the dose to ablate endogenous spermatogenesis (Hirayanagi et al., 2015). Thus, the success of xenotransplantation was not dependent on busulfan-induced immune suppression.

To distinguish transplanted spermatogonia from endogenous spermatogenesis that might recover after busulfan treatment, in this study, we utilized two markers, DDX4 and NuMA, to quantify human spermatogonia in mouse recipients. DDX4 (also called VASA), expressed only in germ cells (Castrillon et al., 2000; Tanaka et al., 2000), was used to detect spermatogonia, while NuMA (Merdes and Cleveland, 1998; Taimen et al., 2004), verified specifically for human cells, efficiently recognized human spermatogonia in mouse testes. The combination of two markers can, to some extent, facilitate the reliable detection of human spermatogonia *in vivo*. We found all DDX4 germ cells were NuMA positive, thus confirming their human origin. These DDX4+ germ cells were also co-stained with a spermatogonial marker, PLZF, suggesting that they are undifferentiated spermatogonia. These findings are consistent with previous studies (Nagano et al., 2002; Sadri-Ardekani et al., 2009; Sadri-Ardekani et al., 2011), indicating that only human spermatogonia survived long-term after xenotransplantation but could not go beyond the spermatogonial stage in mouse testes. Although xenotransplanted cell populations may potentially be contaminated with human testicular somatic cells (e.g., Sertoli cells) during spermatogonial collection from testes or SLC differentiation, very few NuMA positive cells were DDX4 negative. Therefore, we conclude that the human germ cell engraftment was mainly supported by the mouse testis microenvironment rather than by co-transplanted human testicular somatic cells.

The last 2 decades have witnessed remarkable technical advances to reconstitute germ cell development from PSCs *in vitro* (Kee et al., 2009; Hayashi et al., 2011; Easley et al., 2012; Irie et al., 2015; Sasaki et al., 2015; Zhou et al., 2016; Zhao et al., 2018; Gell et al., 2020). These approaches not only offer powerful tools to probe the fundamental regulatory mechanisms of mammalian reproduction, but also open a door for developing replacement therapy of male infertility. Modified from an approach that was originally developed by Easley *et al.* (Easley et al., 2012), we developed a feeder- and xeno-free culture condition that enabled robust derivation of PLZF+ SLCs from human PSCs, including both ESCs we used in this study and patient-derived iPSCs (Zhao et al., 2018). These *in vitro* derived SLCs displayed key features of spermatogonia, with upregulated germline genes and specific epigenetic imprinting patterns (Easley et al., 2012; Zhao et al., 2018). In this study, using a combination of EpCAM and TNAP staining, we further developed a strategy to remove residual ESCs from SLCs. We found that these EpCAM-/TNAP+ cells highly expressed spermatogonial genes, including PLZF, INTERGRINα6, TKTL1, and DMRT3, but were negative for PSC marker TRA-1-81. Upon transplantation, none of these EpCAM-/TNAP+ cells formed teratoma. More importantly, similar to *in vivo* developed human spermatogonia, EpCAM-/TNAP+ SLCs from human ESCs were able to go through the homing process. They were successfully recognized by Sertoli cells in the murine recipients and migrated to the basement membrane of seminiferous tubules of both immunocompromised nude mice and *Kit* ^*w/w-v*^ recipients. Our study thus functionally confirmed the cell identity of these EpCAM-/TNAP+ as prospermatogonia or undifferentiated spermatogonia and offered a feasible approach to study germ cell development with a PSC differentiation platform. In addition, the similar SLC derivation procedure can be applied to patient-derived iPSCs to understand the underlying causes and pathological development of male infertility. Although previous reports have shown minimal immune rejection upon transplantation of syngeneic iPSC-derived cells or tissues (Araki et al., 2013; Guha et al., 2013), it remains as a debatable topic whether PSC-derived cells evoke immune responses differently from those developed *in vivo* (de Almeida et al., 2013; Liu et al., 2017). Our study demonstrated negligible immunogenicity of human PSC-derived SLCs in mouse testes, thereby providing strong evidence to support the application of autologous iPSC derivatives for future therapeutic purposes.

## Data Availability

The original contributions presented in the study are included in the article/Supplementary Material, further inquiries can be directed to the corresponding authors.
