# Supplementary figures and images for: Xenotransplantation of Human Spermatogonia Into Various Mouse Recipient Models
Source: Front Cell Dev Biol. 2022 May 23;10:883314. doi: 10.3389/fcell.2022.883314 (PMC9168328; doi:10.3389/fcell.2022.883314)

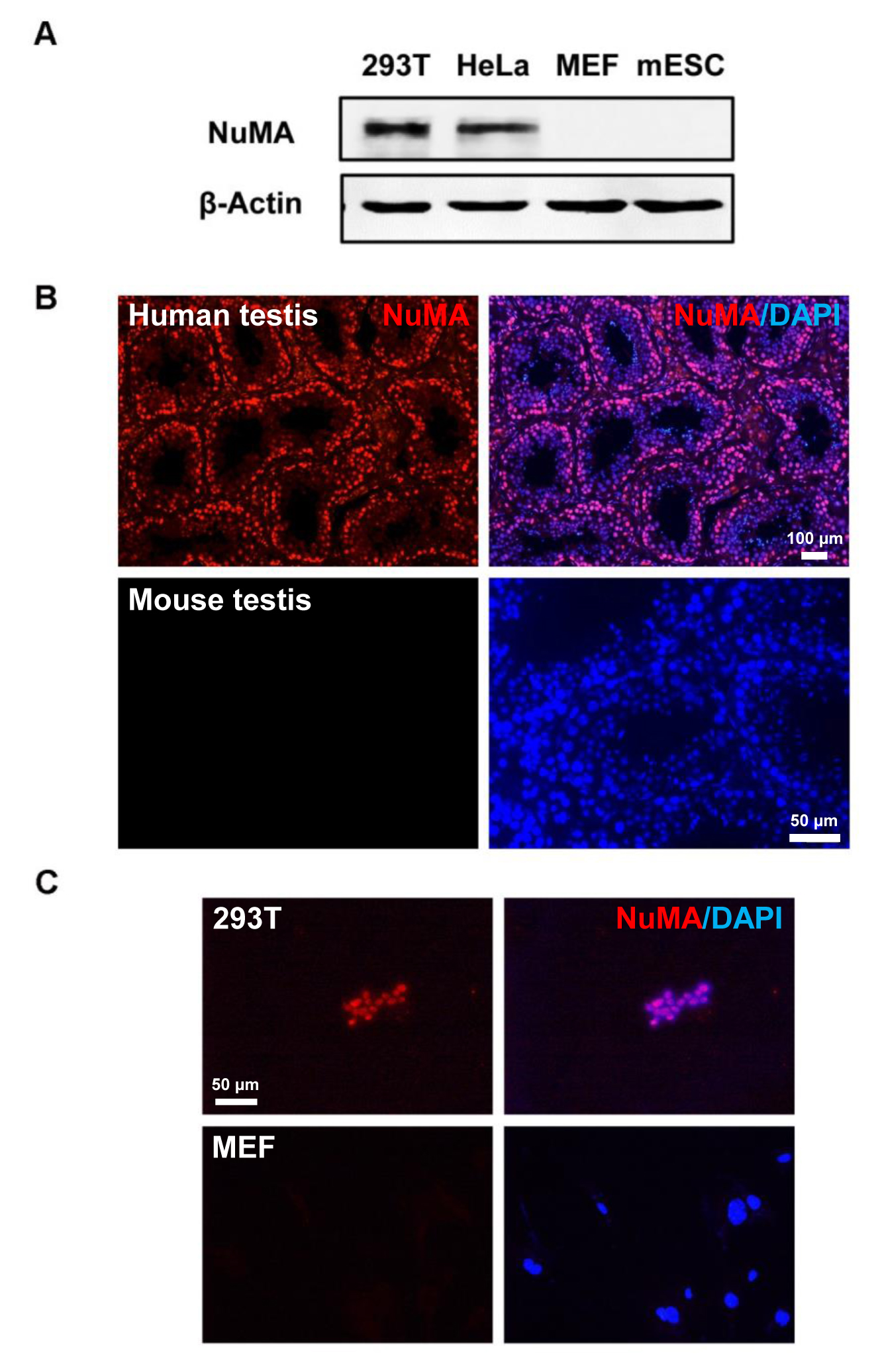

Supplement: Supplementary file 1 [file Image3.JPEG]

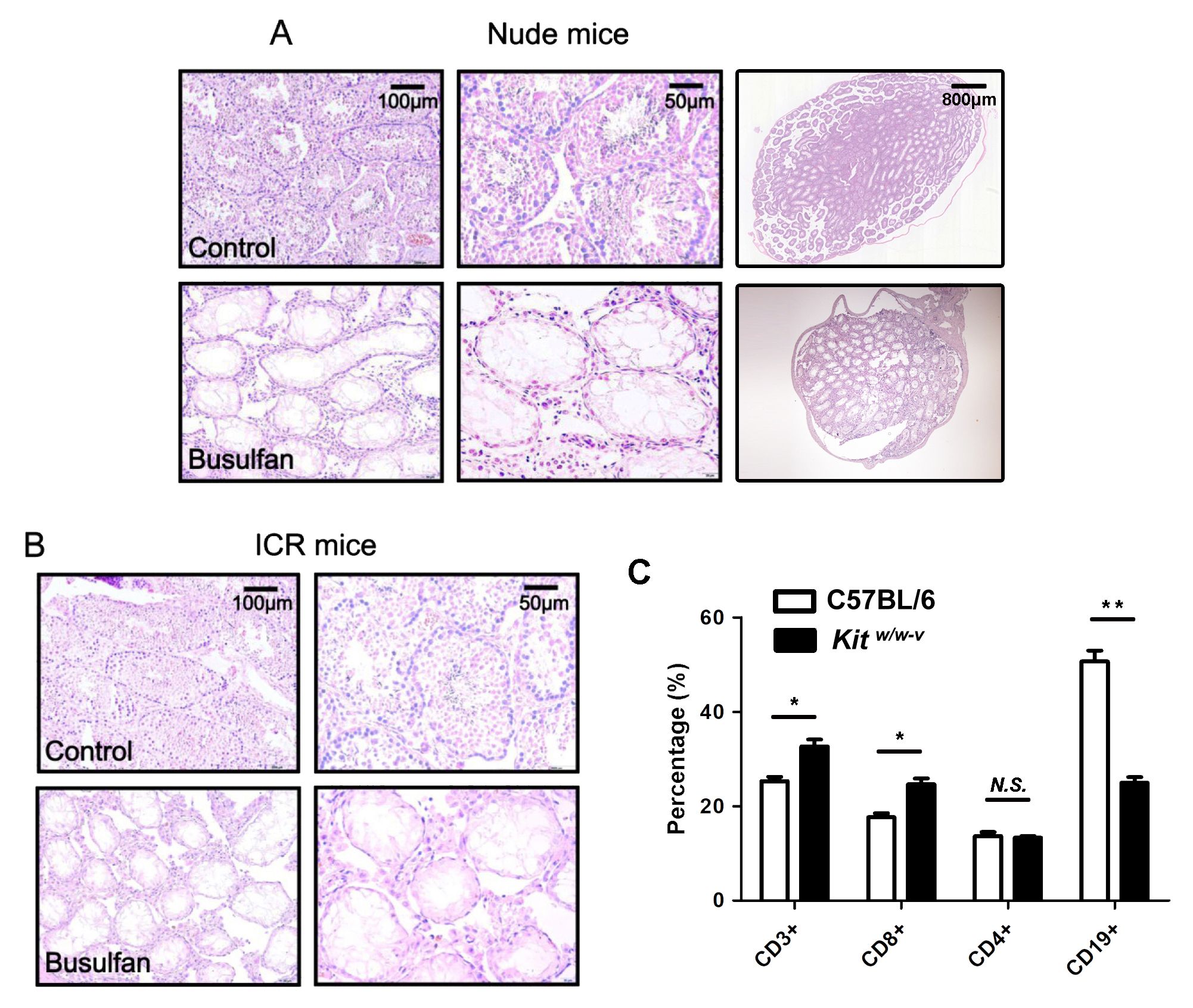

Supplement: Supplementary file 2 [file Image1.JPEG]

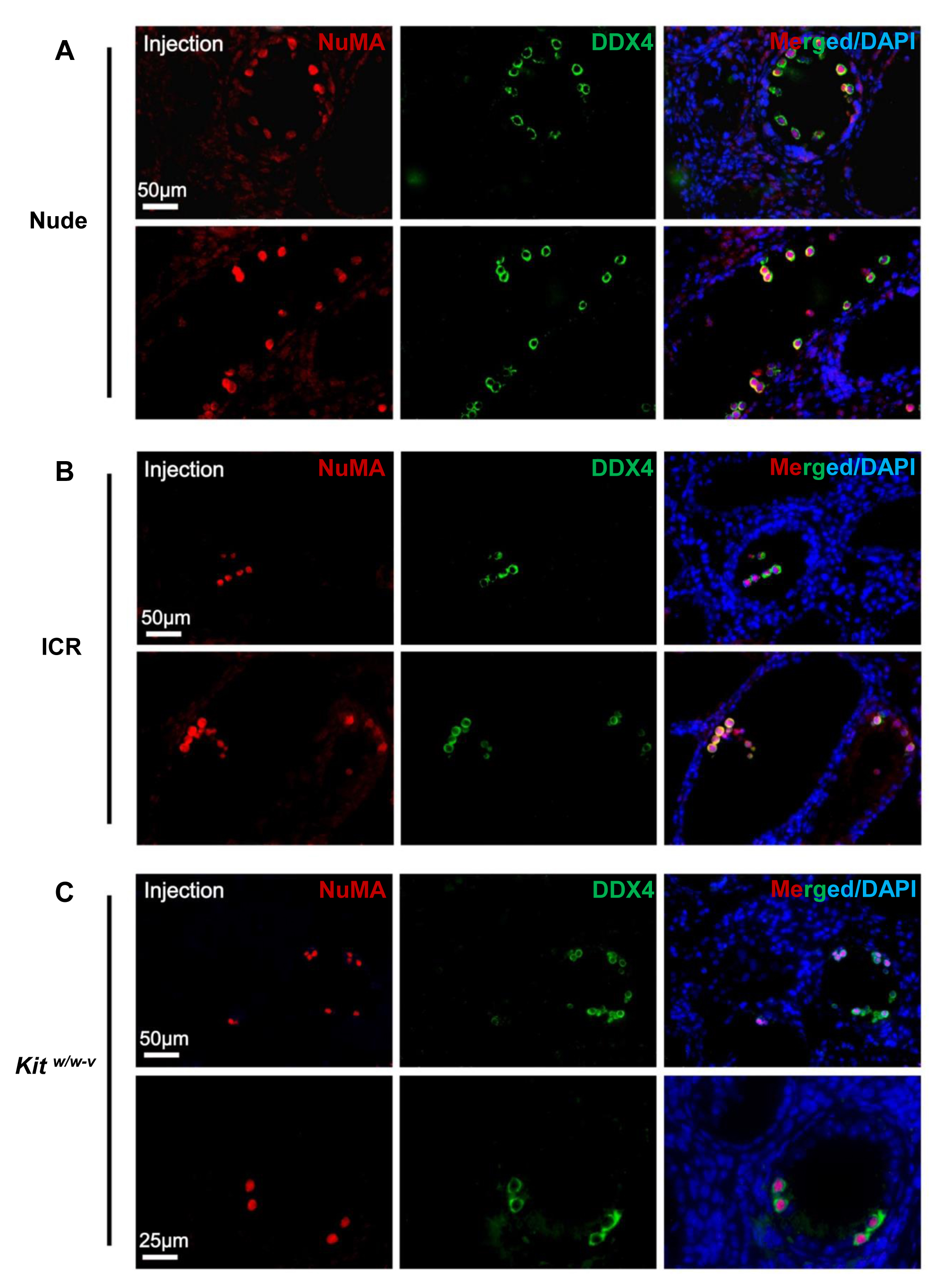

Supplement: Supplementary file 3 [file Image4.JPEG]

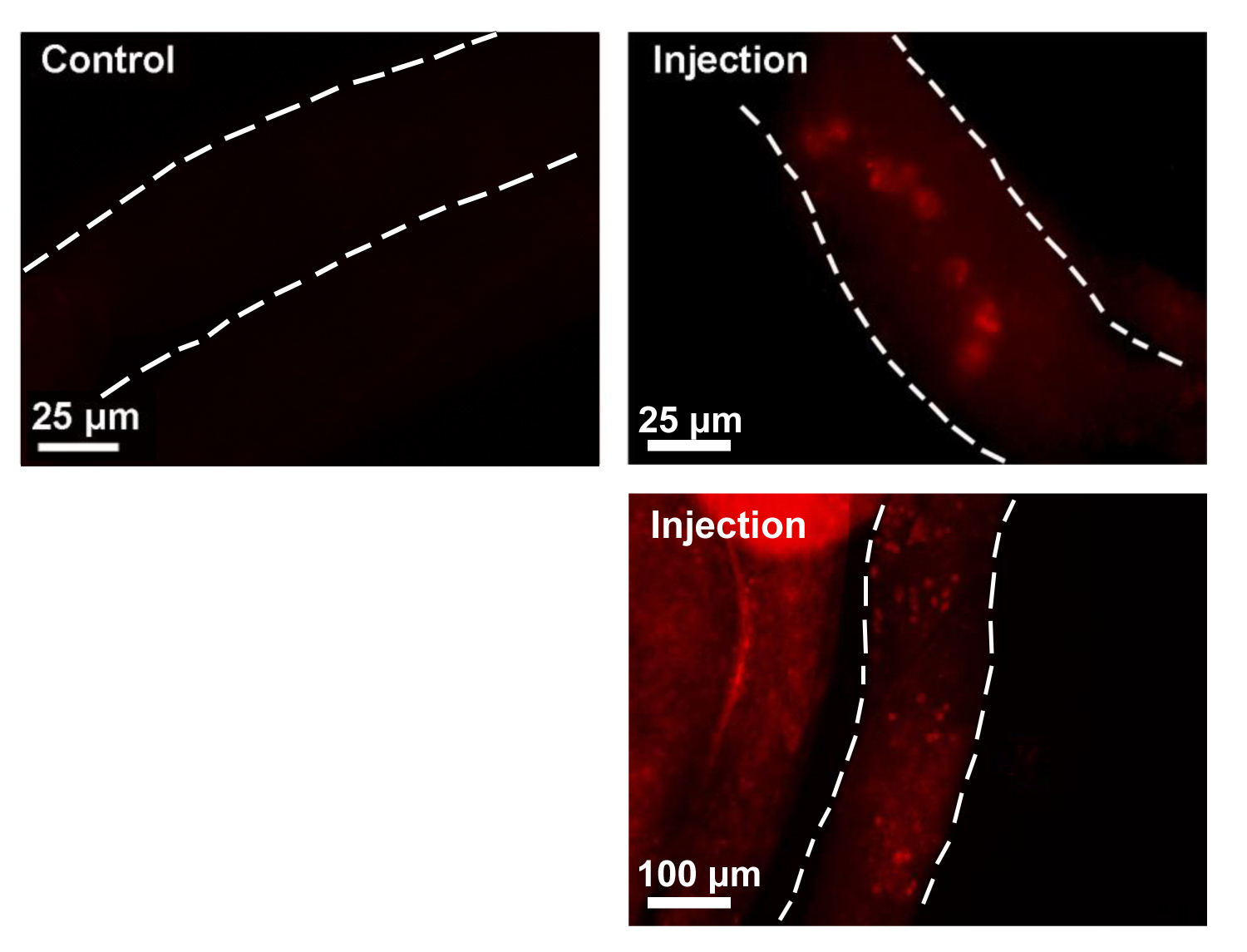

Supplement: Supplementary file 4 [file Image7.JPEG]

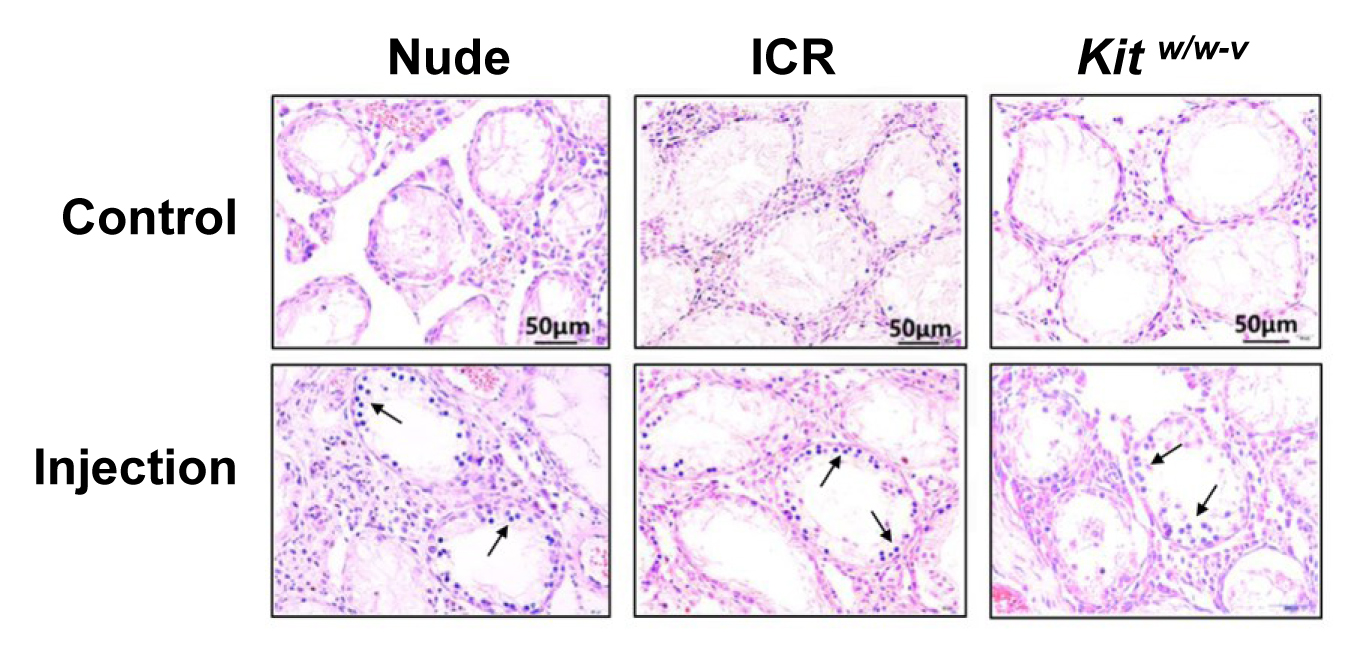

Supplement: Supplementary file 5 [file Image2.JPEG]

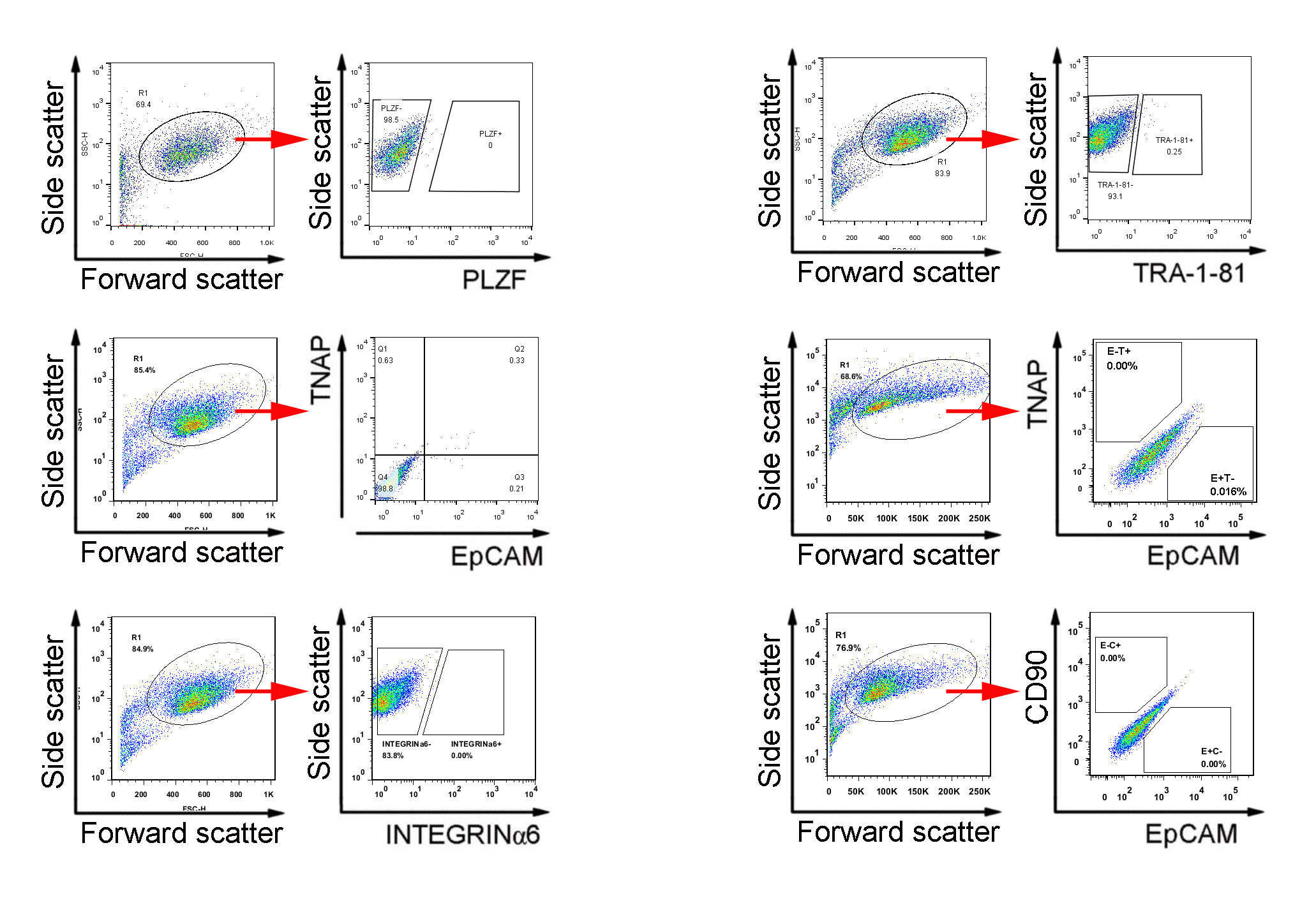

Supplement: Supplementary file 6 [file Image5.JPEG]

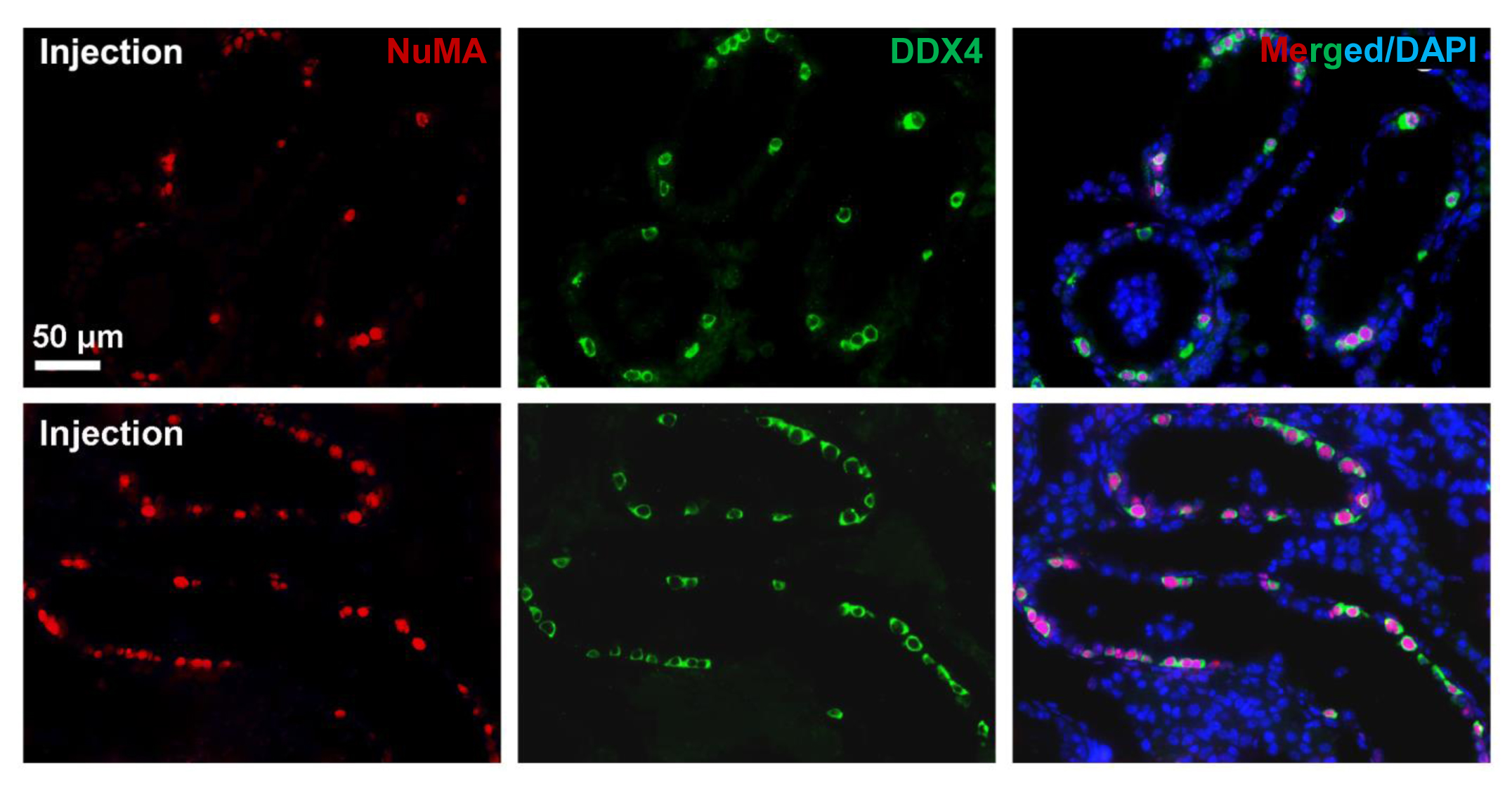

Supplement: Supplementary file 10 [file Image6.JPEG]
