# Supplementary material for: Xenotransplantation of Human Spermatogonia Into Various Mouse Recipient Models
Source: Front Cell Dev Biol. 2022 May 23;10:883314. doi: 10.3389/fcell.2022.883314 (PMC9168328; doi:10.3389/fcell.2022.883314)
Supplement: Supplementary file 8 [file DataSheet2.docx]

**Supplementary Information**

**Supplementary Materials and Methods**

***Whole-Mount Immunofluorescent Staining***

Intact seminiferous tubules were collected from mouse recipient testes at six weeks after transplantation. Donor-derived human cells were detected by whole-mount immunofluorescent staining with a NuMA antibody (Novus Biologicals, NB100-74636), as described previously^[75](#_ENREF_75)^. Briefly, intact seminiferous tubules were dehydrated in methanol concentration gradients prior to incubation in methanol: DMSO: H_2_O_2_ (4:1:1), for less than 2 hours. All dehydration, rehydration, blocking, and washing steps were performed in 12-μm pore size Transwell baskets (Corning Life Sciences). The NuMA antibody was used at a 1:500 dilutions and detected with goat anti-rabbit IgG conjugated with AlexaFluor 568 (Invitrogen). Samples were visualized with a Leica fluorescent microscope.

***Western Blotting***

Total proteins were isolated using RIPA lysis buffer (Beyotime Biotechnology), subjected to SDS-PAGE, transferred to PVDF membranes, and incubated with primary antibodies, NuMA (Novus Biologicals, NB100-74636) and β-ACTIN (Santa Cruz, sc-10731). Quantification of target protein levels was performed using the ECL detection system and Quantity One software (Bio-Rad).

**Supplementary Figure S1. Prepare and characterize mouse recipients for xenotransplantation. (A-B)** Busulfan treatment successfully ablated the endogenous germ cells from nude and ICR testes. Histology was performed at six weeks post busulfan treatment. Mice at the same age without injection were used as controls. (**C**) The percentages of CD3+, CD8+, and CD4+ T lymphocytes, as well as CD19+ B lymphocytes from peripheral blood from *Kit^w/w-v^* and C57BL/6 mice were determined with flow cytometry. Data represent as the mean ± SEM (n=3). *: *p* < 0.05; **: *p* < 0.01.

**Supplementary Figure S2. Nude, ICR, and *Kit^w/w-v^* testes were engrafted with human spermatogonia.** Histology was performed at six weeks post-transplantation with human germ cells. The contralateral testes in the same mice with PBS injection were used as controls.

**Supplementary Figure S3. NuMA antibody recognizes human but not mouse cells.** (**A**) Western blotting analyses with a NuMA antibody on human cell lines (293T and HeLa) and mouse cell lines. MEF: mouse embryonic fibroblast; mESC: mouse embryonic stem cell. (**B**) IHF assays on human and mouse testes with a NuMA antibody, countered with DAPI. (**C**) IF assays of human (293T) and mouse (MEF) cells with a NuMA (red) antibody and DAPI (blue) staining.

**Supplementary Figure S4. Nude, ICR, and *Kit^w/w-v^* testes were engrafted with human spermatogonia.** (**A-C**) IHF assays were performed on nude, ICR, and *Kit^w/w-v^* testes six weeks post-transplantation of human spermatogonia with NuMA and DDX4 antibodies, countered with DAPI. Results showed engraftments of human germ cells in all three recipient mouse models.

**Supplementary Figure S5. Negative gating controls for flow cytometry analyses (Figure 4) on human PSC-derived SLCs.**

**Supplementary Figure S6.** ***Kit^w/w-v^* testes were engrafted with human PSC-derived SLCs.** IHF assays were performed on *Kit^w/w-v^* testes six weeks post transplantation of PSC-derived SLCs with NuMA and DDX4 antibodies, countered with DAPI. Both upper and lower panels showed robust engraftments of human germ cells in *Kit^w/w-v^* testes.

**Supplementary Figure S7. Whole-mount IF with a NuMA antibody on *Kit^w/w-v^* testes transplanted with human SLCs.** Dotted lines show the boarder of seminiferous tubules. Intact seminiferous tubules were collected from mouse recipient testes six weeks after transplantation. Donor-derived human cells were detected by whole-mount immunofluorescent staining with a NuMA antibody (Novus Biologicals, NB100-74636). Samples were visualized with a Leica fluorescent microscope.

**Supplementary Table 1. Primers used for real-time PCR analyses**
