# Supplementary material for: Xenotransplantation of Human Spermatogonia Into Various Mouse Recipient Models
Source: Front Cell Dev Biol. 2022 May 23;10:883314. doi: 10.3389/fcell.2022.883314 (PMC9168328; doi:10.3389/fcell.2022.883314)

**Materials and Methods**

Overall experimental design was shown below. Briefly, we used two types of human spermatogonia for xenotransplantation, one from biopsied human testes, the other from SLCs collected from differentiated PSCs. Three types of recipients were used, including immune-deficient nude mice, immune-competent ICR mice, and genetically infertile *Kit^w/w-v^* mice, as listed on the right of the graph.


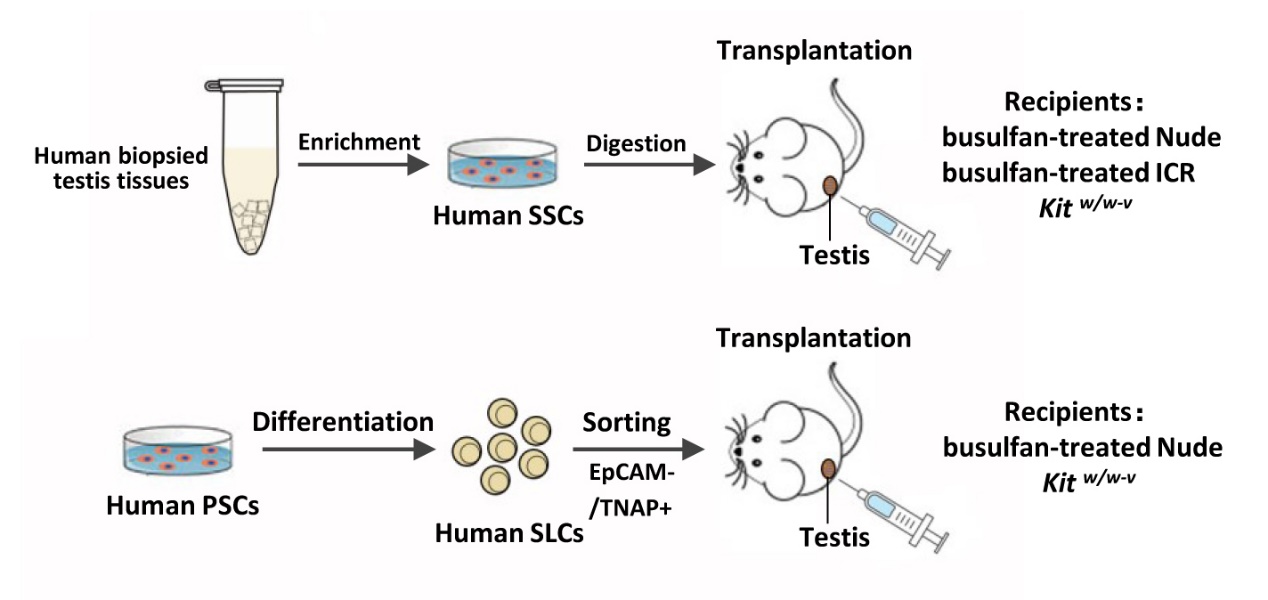

Supplement: Supplementary file 9 [file DataSheet1.docx]
